# The association between fear of disease progression and financial toxicity in patients with chronic osteomyelitis: a cross-sectional study

**DOI:** 10.3389/fpsyg.2026.1785403

**Published:** 2026-03-13

**Authors:** Qiyuan Sun, Jiaxin Yu, Rui Zhang, Junxue Wu, Mengying Yu, Lu Chen, Xiaoqin Li, Yuanyuan Liao, Min Tan

**Affiliations:** 1 Second Department of Surgery, Affiliated Hospital of North Sichuan Medical College, Nanchong, China; 2 School of Nursing, North Sichuan Medical College, Nanchong, China; 3 Department of Orthopedics, Affiliated Hospital of North Sichuan Medical College, Nanchong, China; 4 Nursing Department, Affiliated Hospital of North Sichuan Medical College, Nanchong, China

**Keywords:** chronic osteomyelitis, cross-sectional studies, fear of disease progression, financial toxicity, psychological distress

## Abstract

Chronic osteomyelitis (COM) is a persistent and refractory bone infection associated with substantial physical, psychological, and economic burdens. However, evidence linking financial toxicity (FT) to fear of disease progression (FoP) in patients with COM remains scarce. In this cross-sectional study, we recruited 214 patients with COM from a tertiary hospital in Nanchong, China, between June 2024 and June 2025. Participants completed electronic questionnaires assessing demographic/clinical characteristics, FoP (Fear of Progression Questionnaire-Short Form), and FT (Comprehensive Score for Financial Toxicity). The mean FoP score was 38.31 ± 7.18. Notably, 77.1% (165/214) of patients exhibited clinically relevant levels of FoP (score ≥ 34), indicating a high prevalence of psychological distress. Spearman’s rank correlation was observed between FoP and COST scores (*r*_s_ = −0.599, *p* < 0.001), indicating that higher financial toxicity (lower COST scores) was associated with higher FoP. Hierarchical multiple linear regression identified longer disease duration, higher pain intensity, the presence of complications, and lower COST scores (*B* = −0.279, *p* < 0.001) as independent factors associated with elevated FoP (all *p* < 0.05). These findings underscore a critical interplay between economic and psychological distress in COM and highlight the urgent need for integrated clinical strategies that combine psychosocial support with financial navigation to mitigate FoP and improve patient wellbeing.

## 1 Introduction

Chronic osteomyelitis (COM) is a protracted and difficult-to-treat skeletal infectious disease that poses a serious threat to patients’ physical and mental health. It is characterized by a prolonged disease course and a high recurrence rate, and it often leads to bone destruction, soft-tissue injury, and a spectrum of severe complications, including permanent functional impairment (Glaudemans et al., 2019; Masters et al., 2022). In recent years, the global incidence of chronic osteomyelitis has increased markedly, driven by factors such as population aging, rising rates of diabetes, and trauma (Kremers et al., 2015; Pollard et al., 2006). This burden is particularly pronounced in developing countries, where limited medical resources and economic constraints exacerbate the challenge (Lazzarini et al., 2004; Wirbel and Hermans, 2014). Crucially, the disease’s recurrent nature and prolonged treatment course not only severely impair patients’ physical function and work capacity but also impose a substantial financial burden on their families (Maffulli et al., 2016). Further research shows that the persistent pain, restricted mobility, and diminished quality of life caused by COM impose a substantial physical burden and exert profound psychological effects (Wang et al., 2017; Momodu and Savaliya, 2025).

Fear of progression (FoP) was first introduced by Dankert et al. (2003) and refers to the persistent, excessive worry experienced by patients about disease deterioration or recurrence. This psychological state can exert wide-ranging negative effects on physical, psychological, and social functioning. FoP typically lies between adaptive and maladaptive responses. A moderate level of fear of progression can be viewed as an adaptive reaction that helps patients remain alert to signs of recurrence and maintain a healthy lifestyle (Simard and Savard, 2015). However, when it develops into clinically excessive fear, it may lead to a range of maladaptive behaviors—such as hypervigilance to bodily symptoms, repeated checking, and reassurance seeking—that significantly impair quality of life (Cho and Park, 2017). Studies have shown that excessive FoP can drive patients to adopt negative coping strategies and may even reduce treatment adherence. At present, FoP is recognized as one of the most common forms of psychological distress among patients with cancer and other chronic diseases, such as diabetes (Wang et al., 2022) and heart disease (Xiong et al., 2023). Specifically, among cancer patients, FoP is closely associated with heightened anxiety and depression and reduced treatment adherence (Li et al., 2024); among stroke patients, reducing FoP helps alleviate post-stroke depression (Ning et al., 2025); and among heart failure patients, FoP is strongly negatively correlated with quality of life (Chen et al., 2025). Given its prolonged course, high recurrence rate, and delayed wound healing, COM often brings sustained physical suffering and psychological stress, predisposing patients to fear, unease, and anxiety, which in turn further impair treatment progress and rehabilitation outcomes. Consequently, FoP has become a prevalent and clinically important issue in this population (Olivero and Leombruni, 2024). However, research on the specific mechanisms and impacts of FoP among patients with COM remains limited.

The concept of Financial Toxicity (FT) was first proposed by Zafar et al. (2013) and is defined as the combination of objective material losses and subjective psychological distress experienced by patients during disease treatment. Specifically, FT encompasses a series of objective burdens and subjective difficulties borne by patients and their families, including high medical expenses, significant reductions in income, debt accumulation, and associated psychological stress (Carrera et al., 2018; Zafar and Peppercorn, 2023). At present, research on FT in China mainly focuses on patients with malignant tumors. In contrast, studies conducted abroad adopt a broader perspective: while continuing to examine oncology populations, they have extended to other chronic diseases such as cardiovascular diseases (Sukumar et al., 2023), inflammatory bowel disease (Bloomfeld and Bickston, 2021), and diabetes (Patel, 2025). A large body of research indicates that FT significantly reduces patients’ quality of life and treatment adherence, and may even lead to poorer survival (Hazell et al., 2020; GBD 2023 Disease and Injury and Risk Factor Collaborators, 2025). Taking COM as an example, its prolonged course—often involving multiple surgeries, long-term antibiotic use, and extensive rehabilitation—frequently results in substantial medical costs. Meanwhile, patients commonly experience impaired work capacity and may take leave or leave their jobs because of treatment needs, leading to substantial income loss. These heavy financial burdens heighten stress and adversely affect treatment outcomes (Geraghty and LaPorta, 2019). However, in the field of COM, investigations that specifically address FT remain limited. Existing evidence suggests that economic stress may intensify psychological burden and thereby amplify patients’ fear of disease progression; conversely, excessive fear may drive patients to seek additional medical resources, further aggravating their financial burden—forming a vicious cycle of mutual reinforcement (Zhang et al., 2023; Xu et al., 2024).

However, in this special patient population with COM, the specific pathways and magnitude of the interaction between the two have not been systematically examined. In view of the epidemiological trend of a continued increase in the global prevalence of COM, evidence on the psychological and economic burdens in this group remains scarce. To our knowledge, this is one of the first studies to explicitly quantify the correlation between FoP and FT in the COM population. Therefore, we adopted a cross-sectional design and recruited adult patients with COM in China. The study focuses on the association between FoP and FT, aiming to elucidate the intrinsic link between these dual burdens and to provide an evidence base for the development of targeted clinical psychological and social support strategies in the future.

## 2 Methods

### 2.1 Design and sample

This study employed a cross-sectional design and recruited participants via convenience sampling from the Department of Orthopedics at a tertiary grade-A general hospital in Nanchong, Sichuan Province. Inclusion criteria were: (1) age ≥ 18 years; (2) meeting the diagnostic criteria for chronic osteomyelitis (Wu et al., 2019); (3) voluntary participation with signed informed consent. Exclusion criteria were: (1) comorbid psychiatric disorders or cognitive impairment (verified by medical records and brief screening during enrollment); (2) concomitant chronic pain conditions unrelated to COM (e.g., severe osteoarthritis, fibromyalgia, or neuropathic pain), as distinguished through detailed medical history and clinical evaluation; (3) participation in other similar studies at the same time.

According to Kendall’s estimation method (Bacchetti and Leung, 2002), the required sample size is 5–10 times the number of variables. Based on a literature review, this study included 23 independent variables; therefore, the minimum required sample size ranged from 115 to 230. Allowing for an anticipated 20% rate of invalid questionnaires, the target sample size was expanded to 138–276. Ultimately, 219 questionnaires were collected, meeting the sample size requirement.

### 2.2 Data collection and quality control

Data were collected from June 2024 to June 2025. The survey was administered online via Wenjuanxing (a Chinese online survey platform). Prior to data collection, all researchers received standardized training on participant recruitment and online survey procedures. During enrollment, the study procedures were explained to patients and their family members, and informed consent was obtained; all questionnaires were completed anonymously. The Wenjuanxing system required completion of all items before submission and restricted each WeChat account to a single response. After data collection, the dataset was exported and independently reviewed by two researchers, and questionnaires with obvious logical contradictions or clearly unreliable response patterns were excluded. In total, 219 questionnaires were submitted; after quality checks, 214 valid questionnaires were retained, yielding an effective response rate of 97.7%.

### 2.3 Ethics approval and consent to participate

This study was approved by the Ethics Committee of the Affiliated Hospital of North Sichuan Medical College (2024ER365-1) and conducted in accordance with the Declaration of Helsinki and relevant guidelines and regulations. The informed consent statement was embedded on the first page of the online questionnaire; only participants who confirmed their consent electronically could proceed to complete the survey. Participation was entirely voluntary, and anonymity and confidentiality of all responses were ensured.

## 3 Research instruments

### 3.1 Questionnaire for general information

Based on a literature review and expert guidance, the research team synthesized factors potentially influencing FoP in patients with chronic osteomyelitis and self-designed a structured survey questionnaire (Supplementary File 1). The questionnaire comprised sociodemographic characteristics (sex, age, marital status, educational level, employment status, place of residence, living arrangement, medical payment methods, monthly household income) and disease-related information (disease duration, number of hospitalizations, site of infection, etiology, comorbidities, complications, pain score, and perception of disease recurrence). Pain was assessed using the Visual Analogue Scale (VAS), with 0–3 indicating no/mild pain, 4–6 indicating moderate pain, and 7–10 indicating severe pain.

### 3.2 Fear of progression questionnaire-short form (FoP-Q-SF)

The scale was developed by Mehnert et al. (2006) and consists of 12 items across two dimensions: social/family and physical health. In this study, the Chinese version of the FoP-Q-SF, validated for use in Chinese populations by Wu et al. (2015), was employed. It uses a 5-point Likert format, scored 1–5 from “never” to “always,” yielding a total score of 12–60, with higher scores indicating a greater level of FoP. A total score ≥34 indicates clinically relevant levels of FoP. In this study, the scale’s Cronbach’s *α* was 0.93, indicating excellent internal consistency.

### 3.3 Comprehensive score for financial toxicity (COST)

The scale was developed by de Souza et al. (2014) to assess patients’ perceived financial stress over the past week. In this study, the Chinese version of the COST scale, which was translated and validated by Yu et al. (2017), was employed. It comprises three dimensions—economic expenditures (1 item), economic resources (2 items), and psychosocial responses (8 items)—for a total of 11 items. Items 1, 6, 7, and 11 are positively scored, whereas Items 2, 3, 4, 5, 8, 9, and 10 are reverse scored. The instrument uses a 5-point Likert scale from “not at all” to “very much,” scored 0–4, yielding a total score of 0–44; lower scores indicate greater financial toxicity. In this study, the scale’s Cronbach’s α was 0.91, indicating excellent internal consistency.

### 3.4 Data analysis

Statistical analyses were performed using SPSS Version 27.0. The normality of continuous variables was assessed using the Shapiro–Wilk test and visual inspection of histograms. Normally distributed data (e.g., FoP scores) were presented as mean ± standard deviation (M ± SD). Non-normally distributed data (e.g., COST scores) were presented as median and interquartile range (IQR). Categorical variables were presented as frequencies and percentages. Between-group comparisons were conducted using independent-samples t-tests or one-way analysis of variance (ANOVA). Correlations between variables were examined using Spearman’s rank correlation analysis to account for the ordinal scale properties and wide distribution of the scores. Multiple linear regression models were used to explore associated factors.

To adjust for potential confounders and assess the independent effect of FT, a hierarchical multiple linear regression analysis was performed. Dummy variables were created for categorical predictors, including disease duration (ref: <1 year), pain score (ref: mild), complications (ref: 0–1 type), comorbidities (ref: none or 1), site of infection (ref: upper limb), level of awareness (ref: unfamiliar), household income (ref: <3,000 RMB), and number of hospitalizations (ref: 1 time). In Model 1, key sociodemographic variables (age, gender, education, employment, residence, income, payment) were entered. In Model 2, clinical variables (including all dummy variables) were added. In Model 3, the COST score was entered to examine its incremental explanatory value. Multicollinearity was assessed using the Variance Inflation Factor (VIF). In addition, residual analysis was conducted for the final model to verify the assumptions of normality, linearity, and homoscedasticity; Cook’s distance was used to identify potential influential cases.

## 4 Results

### 4.1 Participant characteristics

A total of 214 patients with chronic osteomyelitis were included, and their sociodemographic and disease-related characteristics are presented in Table 1. The mean age was 50.04 ± 12.98 years (range, 19–81 years), with 73.4% being male (*n* = 157) and 61.7% being urban residents (*n* = 132). Regarding disease characteristics, a disease duration of 1–2 years accounted for 71.5% of patients (*n* = 153), and ≥ 3 hospitalizations accounted for 52.8% (*n* = 113).

**Table 1:** Comparison of fear of disease progression scores in participants with different characteristics (*n* = 214).

Variables	*n* (%)	Scores of FoP (M ± SD)	*t*/F	*p*-value
Gender			0.034^a^	0.973
Male	157 (73.4)	38.32 ± 7.00		
Female	57 (26.6)	38.28 ± 7.73		
Age			1.077^b^	0.343
<30 years old	29 (13.6)	39.14 ± 7.95		
30–50 years old	79 (36.9)	37.38 ± 6.74		
>50 years old	106 (49.5)	38.77 ± 7.28		
Marital status			0.662^b^	0.577
Unmarried	28 (13.1)	39.89 ± 7.97		
Married	155 (72.4)	38.18 ± 6.98		
Divorced	20 (9.4)	37.95 ± 6.35		
Widowed	11 (5.1)	36.73 ± 9.49		
Education			1.621^b^	0.170
Elementary school and below	74 (34.6)	39.35 ± 7.14		
Junior high school	84 (39.3)	38.62 ± 6.78		
High school/Vocational school	34 (15.9)	37.03 ± 7.98		
Junior college	11 (5.1)	36.64 ± 7.37		
Bachelor’s degree or above	11 (5.1)	34.55 ± 6.89		
Employment status			–2.831^a^	0.005**
Employed	84 (39.3)	36.61 ± 7.25		
Unemployed/Retired	130 (60.7)	39.41 ± 6.95		
Place of residence			-1.586^a^	0.114
Urban	132 (61.7)	37.70 ± 6.79		
Rural	82 (38.3)	39.29 ± 7.71		
Living arrangements			-0.215^a^	0.830
Live alone	28 (13.1)	38.04 ± 7.82		
Living with others	186 (86.9)	38.35 ± 7.10		
Medical payment methods			2.354^b^	0.097
Employee Medical Insurance	54 (25.2)	36.74 ± 7.14		
Urban and Rural Medical Insurance	154 (72.0)	38.71 ± 7.22		
Out-of-Pocket	6 (2.8)	42.00 ± 4.15		
Monthly household income			5.253^b^	0.002**
<3,000 RMB	69 (32.2)	40.00 ± 7.35		
3,000–5,000 RMB	110 (51.4)	38.19 ± 6.82		
5,000–8,000 RMB	31 (14.5)	36.32 ± 6.92		
>8,000 RMB	4 (1.9)	27.75 ± 3.78		
Disease duration			26.035^b^	<0.001**
<1 year	9 (4.2)	27.67 ± 8.40		
1–2 years	153 (71.5)	37.42 ± 6.40		
>3 years	52 (24.3)	42.77 ± 6.30		
Number of hospitalizations			12.998^b^	<0.001**
1 time	55 (25.7)	34.89 ± 8.17		
2 times	46 (21.5)	37.20 ± 7.40		
3 times or more	113 (52.8)	40.42 ± 5.76		
Site of infection			4.020^b^	0.019*
Upper limb	12 (5.6)	33.33 ± 4.98		
Lower limbs	154 (72.0)	38.97 ± 7.25		
Others	48 (22.4)	37.42 ± 6.94		
Etiology			1.547^b^	0.190
Open fracture	61 (28.5)	37.03 ± 7.61		
Postoperative infection	65 (30.4)	39.52 ± 6.31		
Blood-borne infection	3 (1.4)	40.67 ± 10.41		
Diabetic foot infection	20 (9.3)	40.20 ± 8.11		
Others	65 (30.4)	37.60 ± 7.06		
History of amputation			2.648^a^	0.009**
Yes	10 (4.7)	44.10 ± 3.78		
No	204 (95.3)	38.02 ± 7.20		
Pain score (VAS)			32.579^b^	<0.001**
0–3 points (Painless/Mild)	97 (45.3)	34.72 ± 7.42		
4–6 points (Moderate)	101 (47.2)	40.63 ± 5.43		
7–10 points (Severe)	16 (7.5)	45.38 ± 3.38		
Comorbidities			34.751^b^	<0.001**
None or 1 type	43 (20.1)	31.05 ± 6.79		
2–3 types	91 (42.5)	38.22 ± 6.46		
4–5 types	62 (29.0)	41.73 ± 4.73		
≥6 types	18 (8.4)	44.33 ± 4.09		
Complications			59.874^b^	<0.001**
None or 1 type	16 (7.5)	27.69 ± 5.59		
2–3 types	72 (33.6)	34.10 ± 6.38		
4–5 types	103 (48.1)	41.46 ± 4.72		
≥6 types	23 (10.8)	44.78 ± 3.64		
Level of awareness of disease recurrence			4.820^b^	<0.001**
Completely unfamiliar	12 (5.6)	39.42 ± 8.19		
Somewhat familiar	119 (55.6)	38.08 ± 7.33		
Generally familiar	55 (25.7)	40.33 ± 6.35		
Fairly familiar	24 (11.2)	36.25 ± 5.58		
Very familiar	4 (1.9)	26.25 ± 5.50		

FoP = Fear of disease progression; a indicates *t* value; b indicates *F* value. Comorbidities: Diabetes, hypertension, chronic kidney disease, malnutrition, immune system diseases, anemia, and cardiovascular, cerebrovascular, respiratory, or liver diseases. Complications: Pathological fracture, bone or soft tissue defect, joint stiffness, sinus tract formation, functional impairment, limb deformity, muscle atrophy, noninfectious arthritis, and amputation. *p < 0.05, **p < 0.01.

Univariate analyses (Table 1) showed that employment status, monthly household income, disease duration, number of hospitalizations, site of infection, history of amputation, pain score, comorbidities, complications, and perception of disease recurrence were all significantly associated with FoP scores (*p* < 0.05).

### 4.2 Descriptive results for participants in FoP and COST

Based on the normality test and visual inspection of histograms, FoP scores were approximately normally distributed, while COST scores showed a skewed distribution. FoP and COST scores for patients with chronic osteomyelitis are shown in Tables 2, 3. The total FoP score was 38.31 ± 7.18, with physical health scoring 19.63 ± 3.82 and social/family scoring 18.88 ± 4.26. In total, 165 patients (77.1%) had an FoP total score ≥34, indicating clinically elevated levels of FoP. In addition, the COST total score was 8.50 (IQR: 4.00–14.00). Given the scoring range (0–44), this score falls into the lower range of the scale, indicating a severe level of financial toxicity among the participants (noting that lower COST scores indicate greater toxicity). The median scores for each dimension were: economic expenditures 0.00 (IQR: 0.00–1.00), economic resources 2.00 (IQR: 1.00–3.25), and psychosocial responses 6.00 (IQR: 2.00–9.00).

**Table 2:** Total scores and dimension scores of FoP in patients with chronic osteomyelitis (*n* = 214).

Variable	Number of items	M ± SD
FoP	12	38.31 ± 7.18
Physical health	6	19.63 ± 3.82
Social/family	6	18.88 ± 4.26

**Table 3:** Total scores and dimension scores of COST in patients with chronic osteomyelitis (*n* = 214).

Variable	Number of items	Median (P_25_, P_75_)
COST	11	8.50 (4.00, 14.00)
Economic expenditures	1	0.00 (0.00, 1.00)
Economic resources	2	2.00 (1.00, 3.25)
Psychosocial responses	8	6.00 (2.00, 9.00)

### 4.3 Correlation analysis between FoP and COST in patients with chronic osteomyelitis

Considering the wide distribution and ordinal nature of the scores, Spearman’s rank correlation analysis (Table 4) was employed to examine the associations. The results revealed a significant negative correlation between FoP and the total COST score (*r*_s_ = −0.599, *p* < 0.001 for total scores). This indicates that patients with lower COST scores (i.e., greater financial toxicity) experienced significantly higher levels of FoP. Likewise, the sub-dimensions of FoP and COST also showed statistically significant negative correlations (all *p* < 0.001).

**Table 4:** Correlation analysis between FoP and COST in patients with chronic osteomyelitis.

Variables (Dimensions)	FoP	Physical health	Social/family	COST	Economic expenditures	Economic resources	Psychosocial responses
FoP	1						
Physical health	0.914**	1					
Social/family	0.926**	0.747**	1				
COST	−0.599**	−0.575**	−0.567**	1			
Economic expenditures	−0.315**	−0.282**	−0.296**	0.658**	1		
Economic resources	−0.517**	−0.519**	−0.488**	0.897**	0.501**	1	
Psychosocial responses	−0.607**	−0.575**	−0.579**	0.986**	0.597**	0.836**	1

FoP = Fear of disease progression; COST = Comprehensive Score for Financial Toxicity. Values represent Spearman’s rank correlation coefficients (*r*_s_). **p < 0.01.

### 4.4 Hierarchical regression analysis of factors influencing FoP

Table 5 presents the results of the hierarchical regression analysis. Model 1 (sociodemographic variables) explained 9.5% of the variance in FoP (Δ*R*^2^ = 0.095, *p* = 0.014). Model 2 (clinical variables) significantly improved the model, adding 50.4% to the explanatory power (Δ*R*^2^ = 0.504, *p* < 0.001). In Model 3, the addition of COST accounted for an additional 3.0% of the variance (Δ*R*^2^ = 0.030, *p* < 0.001). The final model explained a total of 62.9% of the variance (*F* (28, 185) = 11.212, *p* < 0.001).

**Table 5:** Hierarchical multiple regression analysis of factors associated with FoP (Final Model 3).

Variables	Model 1			Model 2			Model 3		
	*B*	SE	*β*	*B*	SE	*β*	*B*	SE	*β*
Step 1: Sociodemographic variables									
Age	−0.980	0.844	−0.097	−0.614	0.623	−0.061	−0.215	0.610	−0.021
Gender	−0.882	1.151	−0.054	0.001	0.859	0.000	0.084	0.829	0.005
Education	−0.569	0.701	−0.086	0.477	0.518	0.072	0.374	0.500	0.056
Employment status	2.533	1.161	0.173*	0.918	0.838	0.063	0.894	0.808	0.061
Place of residence	−0.734	1.483	−0.050	−1.893	1.079	−0.128	−1.532	1.045	−0.104
Medical payment methods	−0.177	1.475	−0.012	0.672	1.087	0.045	0.191	1.056	0.013
Monthly household income (Ref: <3,000 RMB)									
3,000–5,000 RMB	−1.678	1.413	−0.117	−2.175	1.035	−0.152*	−1.196	1.029	−0.083
5,000–8,000 RMB	−2.726	2.198	−0.134	−2.650	1.627	−0.130	−0.340	1.678	−0.017
>8,000 RMB	−10.318	4.011	−0.195*	−3.508	3.169	−0.066	−0.984	3.124	−0.019
Step 2: Clinical variables									
Number of hospitalizations (Ref: 1 time)									
2 times				1.682	1.012	0.096	1.517	0.977	0.087
≥3 times				0.970	0.904	0.068	1.137	0.872	0.079
Disease duration (Ref: <1 year)									
1–2 years				4.080	1.935	0.257*	3.127	1.881	0.197
>3 years				7.103	2.087	0.425**	5.666	2.046	0.339**
Pain score (VAS) (Ref: Mild)									
Moderate				2.328	0.875	0.162**	2.328	0.844	0.162**
Severe				2.699	1.674	0.099	2.302	1.617	0.084
Comorbidities (Ref: None or 1 type)									
4–5 types				3.865	1.420	0.245**	2.450	1.416	0.155
≥6 types				3.932	2.185	0.152	1.956	2.167	0.076
Complications (Ref: 0–1 type)									
2–3 types				4.009	1.114	0.277**	3.263	1.092	0.225**
4–5 types				4.980	0.987	0.347**	4.240	0.971	0.296**
≥6 types				7.037	1.728	0.304**	5.896	1.691	0.255**
Site of infection (Ref: Upper limb)									
Lower limbs				1.912	1.578	0.120	1.996	1.522	0.125
Others				0.291	1.730	0.017	0.614	1.670	0.036
Level of awareness of disease recurrence (Ref: Unfamiliar)									
Somewhat familiar				−0.056	1.698	−0.004	−0.427	1.640	−0.030
Generally familiar				−0.710	1.895	−0.043	−0.860	1.828	−0.052
Fairly familiar				−2.147	2.039	−0.095	−1.488	1.974	−0.066
Very familiar				−2.229	3.281	−0.042	−0.933	3.181	−0.018
History of amputation (Yes)				−0.997	1.806	−0.029	−0.696	1.743	−0.020
Step 3: Financial Toxicity									
COST total score							−0.279	0.072	−0.276**
Model Summary									
*R* ^2^		0.095			0.599			0.629	
Adjusted *R*^2^		0.055			0.541			0.573	
Δ *R*^2^		0.095			0.504			0.030	
*F* value		2.384*			10.289**			11.212**	

B = unstandardized coefficient; SE = standard error; β = standardized coefficient. Variables that were entered into the models but did not show significant main effects in the final presentation are also included in the respective steps to accurately reflect the hierarchical block design. Dummy variables were used for categorical variables as described in the methods. *p < 0.05, **p < 0.01.

In the final model, the COST score was a significant independently associated factor (*B* = −0.279, *p* < 0.001). This negative coefficient confirms that lower COST scores (reflecting greater financial toxicity) were independently associated with higher FoP levels.

Regarding clinical characteristics:

Disease duration: Patients with a duration of >3 years reported significantly higher FoP compared to those with <1 year (*B* = 5.666, *p* = 0.006).

Pain: Compared to mild pain, moderate pain was associated with higher FoP (*B* = 2.328, *p* = 0.006).

Complications: A strong gradient was observed. Compared to 0–1 complication, patients with 2–3 types (*B* = 3.263), 4–5 types (*B* = 4.240), and ≥6 types (*B* = 5.896) reported progressively higher FoP scores (all *p* < 0.01).

Other variables, including age, gender, site of infection, comorbidities, level of awareness, and number of hospitalizations, were not statistically significant in the final multivariable model (*p* > 0.05).

Model diagnostics confirmed the robustness of the final regression. The Durbin-Watson statistic (1.473) indicated the independence of residuals, while visual inspection of residual plots verified that the assumptions of normality and homoscedasticity were adequately met. Furthermore, all Cook’s distance values were below 1, suggesting no influential outliers biased the estimates. Although the VI*F* values for disease duration were slightly elevated (VIF > 5) due to structural multicollinearity inherent in dummy coding, they remained well below the widely accepted critical threshold of 10. Given that the primary predictor, COST (VIF = 2.53), and all other covariates exhibited low VIFs, serious multicollinearity was ruled out, ensuring the stability and reliability of the model.

## 5 Discussion

This study found that both FoP and FT among patients with COM were at or above moderate levels. Hierarchical multiple regression analysis further revealed that the key factors influencing FoP in participants included disease duration, pain severity, complications, and FT. Prior studies (Lu et al., 2022; Klim et al., 2023; Meng, 2023) have shown that the protracted, relapsing nature of COM readily engenders uncertainty about disease progression and prognosis, thereby leading to pronounced negative emotions such as anxiety and depression. Meanwhile, the long-term treatment and care required for the disease unavoidably impose a substantial economic burden (i.e., high financial toxicity denoted by low COST scores) on patients and their families (O’Hara et al., 2024). The interplay between chronic disease and economic strain results in a sustained dual burden on patients’ physical and mental health (Kim et al., 2024). Therefore, a thorough understanding of the current status of FoP and FT, as well as their interrelationship, in this special patient population is crucial for formulating effective clinical interventions and support strategies.

The results of this study show that the total FoP score among participants was 38.31 ± 7.18, which is moderate to high relative to the 60-point scale. This indicates that most participants exhibited elevated FoP, likely attributable to the core features of COM—its protracted, refractory, and relapsing course—which readily trigger profound worries and uncertainty about infection spread, worsening bone destruction, loss of function (particularly when lower-limb involvement impairs ambulation), risk of amputation, and treatment failure. The study also revealed that participants generally experienced high levels of FT (low COST scores), which may be related to the long-term, repeated treatments required (e.g., examinations, antibiotic therapy, surgical debridement, and exercise-based rehabilitation) and the disease’s impact on work capacity (Huang et al., 2023). Notably, 60.7% of the participants were not employed, underscoring the severe impairment of labor capacity and the high risk of illness-induced impoverishment, consistent with the findings of Hah et al. (2021). It is worth noting that educational attainment was generally low (junior high school or below: 73.8%), and disease knowledge was insufficient (Somewhat familiar: 55.6%). Such a lack of disease-related knowledge, together with uncertainty about a long-term illness, can weaken psychological adaptation and foster negative emotions (e.g., anxiety and depression) (Skojec et al., 2025), which may further contribute to elevated levels of FoP.

Financial toxicity is a common phenomenon among patients with COM and may be closely intertwined with FoP, potentially reflecting a bidirectional relationship between financial burden and psychological distress. In this study, Spearman’s rank correlation analysis showed a significant negative correlation between FoP and FT scores (*r*_s_ = −0.599, *p* < 0.001). Given that lower COST scores represent more severe financial toxicity, this negative correlation indicates that patients experiencing greater FT also tended to report higher FoP, highlighting a strong interaction between psychological and economic burdens. Mechanistically, on the one hand, the heavy economic burden itself constitutes a major psychosocial stressor. In this study, 77.1% of patients exhibited clinically relevant levels of FoP, most of whom were married, middle-aged men bearing the primary financial responsibility for their families. Their diminished or lost work capacity may have heightened concerns about the collapse of their breadwinner role and a sense of helplessness regarding the ongoing economic drain caused by the disease. On the other hand, this FT-related stress (role concerns and helplessness) amplifies fear of disease worsening, consistent with the findings of Yang et al. (2025) in breast cancer patients and their family caregivers—that FT, by intensifying fear of recurrence, significantly increases the risk of depression. The substantial psychological burden associated with heightened FoP further reduces treatment adherence (e.g., delaying follow-up visits or reducing medication due to cost concerns), thereby impairing disease control and increasing the risk of recurrence. Disease relapse or progression, in turn, is often followed by a new round of heavier medical expenditures, further exacerbating FT. These findings point to a potential reinforcing loop involving “disease burden—economic stress—psychological fear—disease deterioration”.

The hierarchical multiple linear regression analysis confirmed that disease duration, pain score, complications, and FT were independent factors associated with FoP (*p* < 0.05) after adjusting for sociodemographic and other clinical characteristics. Collinearity diagnostics suggested acceptable multicollinearity among predictors (all VIFs < 5), supporting the stability of the regression estimates. The results indicated a positive association between disease duration and FoP. Patients with longer courses of illness often experience more frequent infectious relapses, persistent pain, multiple surgical interventions, and progressive functional impairment; these problems cumulatively damage daily life, work capacity, and social role identity, which may be linked to intensified fear of disease deterioration (Panteli and Giannoudis, 2016). In addition, the heavy economic burden of long-term treatment, concerns about uncertain therapeutic effects, and pessimistic expectations regarding future health jointly reinforce anxiety about adverse outcomes such as disability and amputation (Geraghty and LaPorta, 2019). Prolonged disease management may also lead to clinician–patient communication fatigue or reduced treatment adherence, fostering feelings of helplessness and loss of control that further amplify FoP. Our findings also showed that pain is closely related to FoP. Severe and persistent pain not only causes marked physical discomfort and activity limitation but also readily triggers negative emotions (e.g., anxiety and depression), potentially reflecting a maladaptive pattern of “pain–negative affect–fear,” which is likely associated with a substantial decline (Hofmann et al., 2017). The presence of complications significantly increased patients’ FoP. Due to disease progression itself or long-term treatment/medication, patients with chronic osteomyelitis may develop complications such as noninfectious arthritis and pathological fractures, and in severe cases may even face the risk of amputation. These potential adverse outcomes heighten concerns about prognosis, thereby escalating FoP (Tharwat and Nassar, 2024). Such complications are often accompanied by persistent pain, limb dysfunction, and reduced mobility, which further restrict daily activities and social participation, lower self-efficacy, and increase psychological burden (Oliver et al., 2018). Notably, FT was verified as a key socioeconomic determinant; its role was consistent with the correlation analysis and was further confirmed in the regression model, aligning with the findings of Geraghty and LaPorta (2019). COM commonly requires long-term treatment, repeated hospitalizations, and substantial out-of-pocket expenses, imposing heavy financial pressure on patients and families and leading to a series of adverse consequences such as heightened anxiety, poorer treatment adherence, and deteriorating quality of life. This suggests that psychosocial support and socioeconomic assistance systems for patients and their families remain inadequate, with needs far from fully met (Oliver et al., 2018). However, it is important to interpret the impact of FT objectively. While the addition of the COST score to the regression model was statistically significant (*p* < 0.001), it only provided an incremental explanatory value of 3.0% (Δ*R*^2^ = 0.030). In contrast, core clinical variables, particularly disease duration, pain severity, and the number of complications, accounted for the vast majority of the variance (50.4%) in FoP. This suggests that while financial toxicity is a crucial compounding factor, the primary drivers of psychological distress in COM patients remain rooted in their direct physical and clinical burdens. Therefore, healthcare professionals should prioritize patients with longer disease duration, higher pain intensity, multiple complications, and heavy financial burden, strengthen health education, encourage adherence to standardized treatment to slow disease progression and prevent complications, and guide families to provide necessary emotional support.

In the univariate analysis, the site of infection was associated with FoP, because of its more direct influence on patients’ mobility and functional recovery. For example, lower-limb osteomyelitis often involves weight-bearing bones such as the femur and tibia, leading to greater pain, joint stiffness, or pathologic fractures that markedly restrict walking, standing, and activities of daily living, thereby severely affecting quality of life and social participation (Hofmann et al., 2017). However, in the multivariable analysis, factors such as site of infection and number of hospitalizations—though significant in the univariate analysis—were not statistically significant in the final model. This suggests a potential mechanism where the effects of these variables on FoP might be indirectly reflected through more direct clinical burdens. For instance, the impact of the site of infection (e.g., lower limb) could potentially overlap with the resulting functional limitations (complications) and pain intensity, which were strong independent factors in our final model. These results suggest that disease duration, pain, complications, and FT are the more direct and stable core determinants.

Based on these findings, integrated clinical strategies addressing both physical and psychosocial burdens may be beneficial. Routine screening for FoP and financial toxicity could be considered for incorporation into standard assessments for patients with COM. For high-risk individuals—such as those with prolonged disease duration, multiple complications, or heavy economic burden—early referral to psychological counseling or social support services might help mitigate distress (Rudolph et al., 2018). In addition, given the low educational attainment observed in our sample, strengthening health education through accessible communication channels is important to mitigate negative emotions driven by information gaps (Sarker et al., 2022). Furthermore, healthcare providers are encouraged to optimize treatment plans to maximize cost-effectiveness and facilitate access to medical assistance resources where possible. Future longitudinal studies are needed to clarify the causal pathways underlying these associations and evaluate the efficacy of such integrated interventions.

## 6 Limitations

This study has several limitations. First, given the cross-sectional design, the findings reflect associations but do not permit causal inference or determination of directionality between FoP and FT. However, sensitivity analyses (Spearman’s rank correlation) confirmed that the observed associations were robust and not driven by outliers or data distribution assumptions. Second, participants were recruited via convenience sampling from a single tertiary hospital, which may limit the generalizability of the results. Although the institution serves as the designated National Osteomyelitis Sub-center and the Regional Medical Center for Northeast Sichuan with a broad referral base, caution is warranted when extrapolating these findings to other settings or populations. Third, outcomes were assessed using self-reported, online questionnaires, which may introduce reporting/recall bias and other measurement-related biases. Future studies should employ longitudinal or prospective multicenter designs, and incorporate more objective or clinician-rated assessments to better clarify causal pathways and reduce potential bias.

## 7 Conclusion

Patients with COM exhibit elevated levels of FoP and FT. These burdens appear to be interrelated and may jointly contribute to poorer physical and psychological wellbeing. Disease duration, pain severity, complications, and FT were identified as significant factors associated with FoP. These findings highlight the need for clinical approaches that address both physical symptoms and psychosocial stressors. Integrating effective pain and symptom management, strategies to reduce FT, and evidence-based psychological interventions may help alleviate FoP and improve patients’ overall quality of life. Future research should employ longitudinal and multicenter designs to clarify causal pathways and support the development of targeted, evidence-informed interventions.

## Data Availability

The raw data supporting the conclusions of this article will be made available by the authors, without undue reservation.
