# Supplementary material for: The association between fear of disease progression and financial toxicity in patients with chronic osteomyelitis: a cross-sectional study
Source: Front Psychol. 2026 Mar 13;17:1785403. doi: 10.3389/fpsyg.2026.1785403 (PMC13023131; doi:10.3389/fpsyg.2026.1785403)
Supplement: Supplementary file 1 [file Data_Sheet_1.PDF]

Supplementary File 1

Article Title: The association between fear of disease progression and financial toxicity in patients with chronic osteomyelitis: a cross-sectional study

Authors: Qiyuan Sun, Jiaxin Yu, Rui Zhang, Junxue Wu, Mengying Yu, Lu Chen, Xiaoqin Li, Yuanyuan Liao*, Min Tan*

Description: This file contains the structured survey questionnaire used in the study, including sociodemographic characteristics, disease-related information, FoP-Q-SF, and COST scales.

Dear Patient,

Thank you very much for participating in this survey. This study aims to understand the clinical condition, treatment history, psychological well-being (such as concerns about disease progression), and financial stress experienced by patients with chronic osteomyelitis. Your feedback is crucial in helping our medical team better understand patient needs and provide data support for improving future treatment plans and patient care measures.

Instructions:

1. This questionnaire covers sociodemographic information, disease-related data, and psychological scales. It is expected to take approximately 10-15 minutes to complete.

2. This survey is anonymous. All data will be kept strictly confidential and used solely for scientific research purposes. Your personal privacy will be protected.

3. There are no right or wrong answers. Please answer based on your true feelings and actual situation over the past week.

Participation is voluntary. By proceeding to answer the questions, you indicate that you understand the purpose of this study and agree to participate.

We sincerely appreciate your time and support!

Part I: Sociodemographic Characteristics

1. Gender: ☐ Male ☐ Female

2. Age: ______ years

3. Marital Status: ☐ Unmarried ☐ Married ☐ Divorced ☐ Widowed

4. Educational Level: ☐ Elementary school or below ☐ Junior high school ☐ High school/Vocational school ☐ Junior college ☐ Bachelor's degree or above

5. Employment Status: ☐ Employed ☐ Unemployed/Retired

6. Residence: ☐ Urban ☐ Rural

7. Living Arrangement: ☐ Live alone ☐ Living with others

8. Medical Payment Method: ☐ Employee Medical Insurance ☐ Urban and Rural Medical Insurance ☐ Out-of-pocket

9. Average Monthly Household Income: ☐ < 3000 RMB ☐ 3000-5000 RMB ☐ 5000-8000 RMB ☐ > 8000 RMB

Part II: Disease-Related Information

10. Disease Duration: ☐ < 1 year ☐ 1-2 years ☐ ≥ 3 years

11. Number of hospitalizations: ☐ 1 time ☐ 2 times ☐ ≥ 3 times

12. Site of Infection:

☐ Upper limbs (e.g., humerus, radius, etc.)

☐ Lower limbs (e.g., femur, tibia, etc.)

☐ Other (please specify) ______

13. Etiology: ☐ Open fracture ☐ Postoperative infection ☐ Blood borne infection ☐ Diabetic foot infection ☐ Other (please specify) _______

14. History of Amputation: ☐ Yes ☐ No

15. Pain Score (VAS): ☐ 0-3 points (Painless/Mild) ☐ 4-6 points (Moderate) ☐ 7-10 points (Severe)

(Note: 0 points: Painless; Below 3 points: Mild pain that is bearable; 4-6 points: Pain interferes with sleep but is still bearable; 7-10 points: Increasingly intense pain that is unbearable.)

16. Comorbidities: (Select all that apply): ☐ Diabetes ☐ Hypertension ☐ Chronic kidney disease ☐ Malnutrition ☐ Immune system diseases ☐ Other (please specify: e.g., cardiovascular diseases, cerebrovascular diseases, respiratory diseases, liver diseases, anemia, etc.) ________

17. Complications: (Select all that apply): ☐ Pathological fracture ☐ Bone defect ☐ Soft tissue defect ☐ Joint stiffness ☐ Other (please specify: e.g., sinus tract formation, functional impairment, limb deformity, muscle atrophy, amputation, noninfectious arthritis, etc.) ________

18. Level of awareness of disease recurrence:

☐ Completely unfamiliar: No knowledge of disease recurrence risks, symptoms, prevention, etc.

☐ Somewhat familiar: Knows only a little about recurrence-related information (e.g., vaguely aware of the possibility of recurrence).

☐ Generally familiar: Understands some recurrence risk factors or symptoms, but not comprehensively.

☐ Fairly familiar: Can list most recurrence-related symptoms, triggers, and basic

prevention methods.

☐ Very familiar: Fully understands recurrence risks, warning signs, coping strategies, and preventive measures.

Fear of Progression Questionnaire-Short Form (*FoP-Q-SF*)

1. I become anxious if I think about my disease progressing.

☐ Never ☐ Rarely ☐ Sometimes ☐ Often ☐ Very often

2. I am nervous prior to doctor's appointments or periodic examinations.

☐ Never ☐ Rarely ☐ Sometimes ☐ Often ☐ Very often

3. I am afraid of pain.

☐ Never ☐ Rarely ☐ Sometimes ☐ Often ☐ Very often

4. I worry about my professional future.

☐ Never ☐ Rarely ☐ Sometimes ☐ Often ☐ Very often

5. When I am anxious, I have physical symptoms (e.g. rapid heartbeat, stomach ache).

☐ Never ☐ Rarely ☐ Sometimes ☐ Often ☐ Very often

6. I am worried that my disease might be hereditary.

☐ Never ☐ Rarely ☐ Sometimes ☐ Often ☐ Very often

7. I am afraid of relying on the help of strangers.

☐ Never ☐ Rarely ☐ Sometimes ☐ Often ☐ Very often

8. I am afraid that I will not be able to pursue my hobbies.

☐ Never ☐ Rarely ☐ Sometimes ☐ Often ☐ Very often

9. I am afraid of severe medical treatments during the course of my disease.

☐ Never ☐ Rarely ☐ Sometimes ☐ Often ☐ Very often

10. I am worried that medications could damage my body.

☐ Never ☐ Rarely ☐ Sometimes ☐ Often ☐ Very often

11. I worry what will happen to my family if something happens to me.

☐ Never ☐ Rarely ☐ Sometimes ☐ Often ☐ Very often

12. I am afraid that I will not be able to work anymore.

☐ Never ☐ Rarely ☐ Sometimes ☐ Often ☐ Very often

Comprehensive Score for Financial Toxicity (*COST*)

1. I know that I have enough money in savings, retirement, or assets to cover the costs of my treatment.

☐ Not at all ☐ A little bit ☐ Somewhat ☐ Quite a bit ☐ Very much

2. My out-of-pocket medical expenses are more than I thought they would be.

☐ Not at all ☐ A little bit ☐ Somewhat ☐ Quite a bit ☐ Very much

3. I worry about the financial problems I will have in the future as a result of my illness or treatment.

☐ Not at all ☐ A little bit ☐ Somewhat ☐ Quite a bit ☐ Very much

4. I feel I have no choice about the amount of money I spend on care.

☐ Not at all ☐ A little bit ☐ Somewhat ☐ Quite a bit ☐ Very much

5. I am frustrated that I cannot work or contribute as much as I usually do.

☐ Not at all ☐ A little bit ☐ Somewhat ☐ Quite a bit ☐ Very much

6. I am satisfied with my current financial situation.

☐ Not at all ☐ A little bit ☐ Somewhat ☐ Quite a bit ☐ Very much

7. I am able to meet my monthly expenses.

☐ Not at all ☐ A little bit ☐ Somewhat ☐ Quite a bit ☐ Very much

8. I feel financially stressed.

☐ Not at all ☐ A little bit ☐ Somewhat ☐ Quite a bit ☐ Very much

9. I am concerned about keeping my job and income, including paid work at home.

☐ Not at all ☐ A little bit ☐ Somewhat ☐ Quite a bit ☐ Very much

10. My illness or treatment has reduced my satisfaction with my present financial situation.

☐ Not at all ☐ A little bit ☐ Somewhat ☐ Quite a bit ☐ Very much

11. I feel in control of my financial situation.

☐ Not at all ☐ A little bit ☐ Somewhat ☐ Quite a bit ☐ Very much
